# Supplementary material for: Identification of Freezing-Responsive microRNAs and Their Targets in Chinese Jujube by Small RNA and Degradome Sequencing
Source: Int J Mol Sci. 2026 Sep 4;27(17):7912. doi: 10.3390/ijms27177912 (PMC13566890; doi:10.3390/ijms27177912)
Supplement: Supplementary file 1 [file ijms-27-07912-s001.zip › Supplementary Material S2/novel_miR_222.pdf]

| | | |
|---|---|---|
| Provisional ID | : | novel_miR_222 |
| Score total | : | 21.4 |
| Score for star read(s) | : | 3.9 |
| Score for read counts | : | 19.5 |
| Score for mfe | : | 0.2 |
| Score for randfold | : | -2.2 |
| Score for cons. seed | : | |
| Total read count | : | 50 |
| Mature read count | : | 49 |
| Loop read count | : | 0 |
| Star read count | : | 1 |

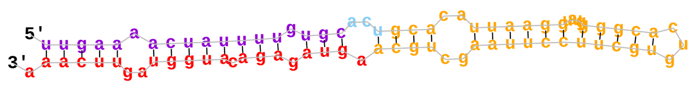


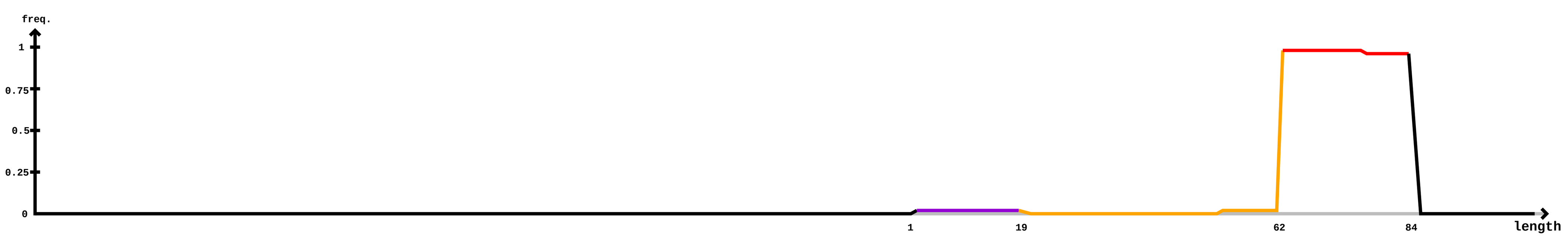


Star Mature

```
5'- uuagauugcuuuauuccuauuguauaguuuuauacuuuucauuuaggguaauaaugagcuuggagcaaugaugcuagcuuaugucaaggggccauuucugacuuuguuacgcuucaucauauugaaucuauauucauugcaacuuuugaaaacuauuuuugugcacugcacauuaagguauggggcacugugcuuccuuaagcugcaaguagagacaugguaguucaaauagagagagcaugcuuucuagu -3'  obs
    uuagauugcuuuauuccuauuguauaguuuuauacuuuucauuuaggguaauaaugagcuuggagcaaugaugcuagcuuaugucaaggggccauuucugacuuuguuacgcuucaucauauugaaucuauauucauugcaacuuuugaaaacuauuuuugugcacugcacauuaagguauggggcacugugcuuccuuaagcugcaaguagagacaugguaguucaaauagagagagcaugcuuucuagu       exp
    .....((((((((........(((((....)))))........))))))))((((((((..((((.((((((((..(((.((...(((((...........))))).)).))).))))))..))..))))..)))))))).....((((((.((((((((.(((..((((..((((((....(((((...)))))))))))..)))).))).))).)))))..))))))(((((((......)))))))..   reads  mm  sample
    .................................................................................................................................................uugaaaacuauuuuugGgc.......................................................................................   1      1   LT-2
    .....................................................................................................................................................................................................uuaagcugcaaguagagacauggu..............................   1      0   LT-1
    ...............................................................................................................................................................................................................aguagagacaugguaguucaaa......................   23     0   LCK-2
    ...............................................................................................................................................................................................................aguagagacaugguaguucaaa......................   26     0   LCK-1
```
